# Aging and Financial Capacity: Cognitive Correlates of an Early Indicator of Functional Decline

**DOI:** 10.1093/geroni/igaa057.1514

**Published:** 2020-12-16

**Authors:** Katherine Wild, Jennifer Marcoe, Nicole Sharma, Mary Siqueland, Nora Mattek, Jason Karlawish, Jeffrey Kaye

**Affiliations:** 1 Oregon Health & Science University, Portland, Oregon, United States; 2 University of Pennsylvania, Philadelphia, Pennsylvania, United States; 3 Layton Alzheimer’s Disease Center, Portland, Oregon, United States

## Abstract

Financial capacity describes the ability to make and carry out sound financial decisions sufficient to meet an individual’s needs for health and well-being. Impairments in financial capacity have been shown to be one of the earliest functional changes in patients with mild cognitive impairment (MCI), the precursor to Alzheimer’s disease and related dementias. Recent developments using online automated monitoring of financial transactions promise a new way to identify the earliest signs of cognitive decline. We examine the feasibility of using secure online technology to link ongoing financial activity monitoring data with other objective measures of function and cognition in a cohort of independent living older adults. To date, 73 older adults (mean age = 76.8, MoCA = 25.9) have enrolled and are participating in a 12-month online financial monitoring program that tracks account activity and generates alerts for unusual or irregular transactions. At baseline participants are administered a battery of neuropsychological tests and the Financial Capacity Instrument (FCI), a measure of financial capacity using tasks of everyday financial activity. Financial monitoring data are collected continuously, and are summarized and reported monthly. Younger participants had more online transactions and higher FCI scores. FCI total score was positively correlated with animal fluency (p < .02), Trails A (p < .03) and B (p < .0001), and visual memory (p < .0008). Number of online transactions in one month was correlated with FCI score, and Trails B (faster time to completion). Lower MoCA scores were associated with higher number of alerts per month.

